# circARL15 Plays a Critical Role in Intervertebral Disc Degeneration by Modulating miR-431-5p/DISC1

**DOI:** 10.3389/fgene.2021.669598

**Published:** 2021-06-21

**Authors:** Hanbang Wang, Yakun Zhu, Le Cao, Ziming Guo, Kai Sun, Wangbao Qiu, Haitao Fan

**Affiliations:** Department of Orthopaedics, Fuyang Hospital of Anhui Medical University, Fuyang, China

**Keywords:** circARL15, miR-431-5p, DISC1, intervertebral disc degeneration, ceRNA

## Abstract

**Background:**

Intervertebral disk degeneration (IDD) is a serious public health problem associated with genetic and environmental factors. However, the pathogenic factors involved and the pathological mechanism of this disease still remain enigmatic.

**Methods:**

The associated microarray was downloaded and further analyzed using statistical software R. The competing endogenous RNA (ceRNA) co-expression network was constructed to measure the meaningful correlated expression of differentially expressed genes. We further measured the expression of circARL15/miR-431-5p/DISC1 in IDD tissues. Cell proliferation and apoptosis were detected in NP cells transfected with a circARL15 overexpression plasmid and miR-431-5p mimics. The expression of DISC1 was detected by immunohistochemistry and Western blot analysis.

**Results:**

Within the ceRNA network, circARL15 is the most differentially expressed circular RNA. circARL15 was down-regulated in IDD and was negatively correlated with miR-431-5p and positively associated with DISC1. miR-431-5p was found to bind directly to circARL15 and DISC1. circARL15 inhibited nucleus pulposus cell apoptosis but promoted nucleus pulposus cell proliferation by targeting the miR-431-5p/DISC1 signaling pathway.

**Conclusion:**

circARL15/miR-431-5p/DISC1 is involved in the pathogenesis of IDD, which might be helpful in determining the diagnostic biomarkers and providing potential therapeutic targets for patients with IDD.

## Introduction

Intervertebral disk degeneration (IDD) is a disease characterized by up-regulated extracellular matrix decomposition and disturbed matrix synthesis (Burke et al., 2019; Sloan et al., 2020). The clinical manifestations of IDD mainly include disk herniation, lumbar instability, and spinal stenosis. IDD is frequently considered to be the chief underlying cause of back pain and is a prevalent disease with high lifetime incidence and associated socioeconomic costs (Andersson, 1999). To date, our limited understanding of the pathophysiological mechanisms of IDD has, in turn, led to a limited understanding of effective diagnostic methods for IDD and insufficient development of novel therapeutic strategies. The etiology of IDD is commonly considered to be multifactorial, with genetic factors contributing to the etiology (Kepler et al., 2013). The newly defined non-coding RNA transcripts (ncRNAs) have been identified recently to be involved in this genetic mechanism (Lan et al., 2016).

Gene expression in the human genome exerts its role in the form of transcripts, over 80% of which are ncRNAs. During recent years, the role of ncRNAs in a variety of physiological or pathological processes has attracted increasing attention (Choudhry and Harris, 2017; Hammond et al., 2017). Circular RNAs (circRNAs) are an important subtype of ncRNAs that are widely distributed in mammals (Yao et al., 2019). With covalently linked ends of a single RNA molecule, ncRNAs appear to be highly stable compared to the linearly associated microRNAs (miRNAs) (Li C. et al., 2019). miRNAs, which are single-stranded and comprise approximately 22–24 nucleotides, can regulate gene degradation or translational repression through binding to microRNA response element (MRE)-targeted mRNA (Iwakawa and Tomari, 2015). Additionally, it has been widely demonstrated that circRNA may harbor specific miRNAs that act as miRNA sponges to regulate gene expression at an epigenetic level (Hansen et al., 2013; Guo et al., 2014). Imbalance of the ceRNA (competing endogenous RNA) intricate network can lead to biological disturbance (Peng et al., 2015). Exploring the interaction of these transcripts would offer a novel perspective for understanding the pathogenesis of many types of pathological process.

Recent studies have revealed that some circRNAs participate in the pathogenesis of IDD, mainly acting as a ceRNA. For instance, circVMA21 has been shown to play a protective role in IDD, possibly by targeting the miR-200c/X-linked inhibitor-of-apoptosis protein signaling pathway (Cheng et al., 2018). The circGRB10/miR-328-5p/ERBB2 signaling pathway has also been shown to contribute to IDD development by regulating nucleus pulposus (NP) cell death (Guo et al., 2018). In IDD, circ4099 regulates extracellular matrix (ECM) synthesis as a compensatory response by reversing the inhibition of Sox9 by miR-616-5p (Wang et al., 2018). However, the specific role of circRNAs in IDD and its pathological mechanism still remains to be elucidated.

In the present study, the differentially expressed genes (DEGs) involved in IDD were identified by analysis of microarray gene-expression data (GSE63492) (Lan et al., 2016). Based on the existed microarray profile, the present study illustrated the molecular profiles underlying ncRNA-correlated patterns in IDD, and investigated the association between these molecules. The ceRNA network was identified and further verified in NP cells. Our findings showed that circARL15 is the mostly differentially expressed circRNA among the ceRNA network and may function as an miR-431-5p sponge to modulate DISC1 expression.

## Materials and Methods

### Samples and Data Acquisition

The GSE67567 dataset was acquired from the National Center of Biotechnology Information (NCBI) Gene Expression Omnibus database^1^, on the basis of GPL15314 Arraystar Human LncRNA and mRNA microarray V2.0 (Agilent_033010 Probe Name version), GPL19449 Exiqon miRCURY LNA microRNA Array, 7th generation REV – hsa, mmu & rno (miRBase v18.0), and GPL19978 Agilent-069978 Arraystar Human CircRNA microarray V1. A total of 10 lumbar disk samples were included for the development of this microarray, including five degenerative nucleus pulposus tissue samples and five samples of healthy lumbar disk.

### Identification of Different Expressions of circRNAs, lncRNAs, miRNAs, and mRNAs

To determine the DEGs, a Limma (linear models for microarray data) package was used, through which data analysis was later performed using an R software package (Kerr, 2003). The statistical significance of the different levels of RNA expression between the two groups was measured according to the *p*-value and false discovery rate (FDR). Fold change ≥ 2.0, *p* < 0.05, and FDR < 0.05 were regarded as the criteria for significant differential expression.

### Construction of the ceRNA Co-expression Network

The ceRNA co-expression network was constructed to measure the meaningful correlated expression of DEGs, as follows: (1) miRcode was used to predict the combined miRNAs of differentially expressed circRNAs; (2) the intersection of the screened results of (1) and differentially expressed miRNAs was analyzed; (3) TargetScan, miRDB, and miRTarBase were used to predict the (2)–mRNA interaction; and (4) the intersection of the screened results of (3) and differentially expressed mRNAs was analyzed to determine the ceRNA.

### Sample Collection

A total of 30 NP specimens were collected in this study, of which 15 were extracted from IDD patients and 15 from normal subjects. The NP specimens of the IDD patients were collected from patients with cervical spondylotic myelopathy, while the normal specimens were extracted from patients with Hirayama disease. These patients had all undergone anterior cervical diskectomy and fusion at Fuyang Hospital of Anhui Medical University. Patients who had undergone radiotherapy or chemotherapy, or had a surgery history, were excluded. The proposed experimental schemes were all approved by the ethics committee of Fuyang Hospital of Anhui Medical University, and informed consent was obtained from each patient enrolled.

### Cell Culture

Following extraction from the intervertebral disk tissues and incubation with 0.25 mg/mL type II collagenase (Sigma-Aldrich, MO, United States) in Dulbecco’s Modified Eagle’s Medium (DMEM) medium (GIBCO, NY, United States) at 37°C for 12 h, NP cells were collected. The isolated cells were further cultured in a mixed DMEM culture medium containing 10% fetal bovine serum, 5 μg/mL gentamicin, and 2 mM glutamine. After the serial subcultivation, we accessed the morphology of the NP cells. Subsequently, the second-passage NP cells were used to perform the following experiments.

### Quantitative Real-Time Polymerase Chain Reaction (PCR)

TRIzol was used to isolate RNAs from the NP specimens of the IDD patients and the control subjects. Using the ABI PRISM7500 system (Applied Biosystems, Carlsbad, CA, United States), the expressions of circRNAs and their targeted mRNAs were quantified. The following primers were used in this study: circARL15 – forward 5′-CAGCTCTTCAGCATCCACAG-3′ and reverse 5′-GATGTTATCAGCCCCTCCAA-3′; GAPDH – for ward 5′-GGGAAACTGTGGCGTGAT-3′ and reverse 5′-GA GTGGGTGTCGCTGTTGA-3′; DISC1 – forward 5′-AGCCGG GATTGCTTACCAC-3′ and reverse 5′-CTGTCGAGCTTCTC ATGTAGC-3′; miR-431-5p – forward 5′-ACGCGTGTCTTGCA GGCCGT-3′ and reverse 5′-ATCCAGTGCAGGGTCCGA GG-3′; RT primer – 5′-GTCGTATCCAGTGCAGGGTCCGAGG TATTCGCACTGGATACGACTGCATG-3′; U6 primer – forward 5′-CTCGCTTCGGCAGCACA-3′ and reverse 5′-AAC GCTTCACGAATTTGCGT-3′.

### Cell Transfection

After culturing in DMEM for 24 h in the cell culture plates, the NP cells were transfected with miR-431-5p mimics, a circARL15 overexpression plasmid, and the corresponding negative control with Lipofectamine 3000 (Invitrogen, Thermo Fisher Scientific). After resting at room temperature, a mixture containing Opti-MEM (Gibco), Lipofectamine 3000, and transfected RNAs was added to each well of the plate. Forty-eight hours after transfection, the cells were collected for further assays. The sequence of miR-431-5p used in this study was 5′-UGUCUUGCAGGCCGUCAUGCA-3′.

### Luciferase and EGFP/RFP Reporter Assay

In the luciferase assay, the wild type (WT) and mutated (MT) sequences of circARL15 and DISC1 were inserted into a firefly luciferase-expressing vector (RiboBio) to reconstruct the recombinant vector. After being seeded into 24-well plates for 24 h, NP cells with an appropriate density were subjected to miR-431 mimics (50 nM) or the negative control using Lipofectamine 3000 (Invitrogen, Thermo Fisher Scientific). Forty-eight hours after transfection, these cells were collected, and the luciferase activity was assessed using the Dual-Luciferase Reporter Assay system (Promega, Madison, WI, United States), according to the manufacturer’s instructions.

To further confirm the direct interaction between circARL15 and DISC1 with miR-431-5p, an EGFP/RFP (enhanced green fluorescent protein/red fluorescent protein) reporter assay was also used. To perform this experiment, NP cells with overexpressed miR-431-5p were co-transfected with vectors containing the circARL15 WT/MT and DISC1WT/MT sequences. The EGFP values of the NP cell groups were measured with an F-4500 fluorescence spectrophotometer (Hitachi, Tokyo, Japan) and normalized according to the RFP values.

### RNA Immunoprecipitation (RIP)

The RIP assay was carried out by using a Magna RIP RNA Binding Protein Immunoprecipitation Kit (Millipore) according to the manufacturer’s instructions. The antibodies against AGO2 and IgG used for the RIP assays were purchased from Abcam (ab5072, rabbit polyclonal antibody, Cambridge, MA, United States).

### Cell Counting Kit-8 (CCK-8) Assay

At the indicated time after transfection, 5.0 mg/mL CCK-8 were added to the plates and cultured with the NP cells for 1 h. After centrifugation, the cells were collected and then incubated with 200 μL of dimethyl sulfoxide. The absorbance of each well was measured at 450 nm using a spectrophotometer (Bio-Rad, Hercules, CA, United States).

### Tunel Assay

TUNEL System kit (BD Biosciences, San Jose, CA, United States) was used to detect the apoptotic DNA fragmentation, according to the manufacturer’s instructions. The apoptotic morphology of each cell sample was observed using a fluorescence microscope.

### Flow Cytometry Assay

A flow cytometry assay was used to monitor the apoptosis level of the NP cells. At the indicated time after transfection, the levels of apoptosis of the NP cells were determined using an Annexin-VFITC/PI Apoptosis Detection Kit (Sigma-Aldrich, St. Louis, MO, United States), according to the manufacturer’s instructions.

### Immunohistochemistry Assay

At the indicated time after transfection, the cells were harvested. After the cells were rinsed in PBS, they were fixed in precooled 4% paraformaldehyde for 15 min. Next, the cells were incubated in a blocking liquid containing goat serum and 0.1% Triton X-100 for 1 h, and then with primary antibody of DISC1 at 4°C overnight. After resting at room temperature, the secondary antibody was used to fix the cells for 1 h at room temperature. After the cells were rinsed in PBS for 30 min, the fluorescent images were captured and analyzed using a microscope (Olympus, Tokyo, Japan) and Image J software, respectively.

### Western Blot Assay

Total protein of the NP cells was extracted using an RIPA lysis buffer (Beyotime, Nantong, China) with a protease inhibitor (Roche, Basel, Switzerland). Following SDS-PAGE (sodium dodecyl sulfate–polyacrylamide gel electrophoresis), and the protein was transferred to a polyvinylidene fluoride membrane and then blocked with 5% skimmed milk at room temperature for 1 h. The protein was then incubated with the primary antibodies of DISC1 (Ab192258, Abcam, 1:1000) and GAPDH (glyceraldehyde-3-phosphate dehydrogenase) (Ab181602, Abcam, 1:1000) overnight at 4°C. On the second day, the membranes were incubated with a secondary antibody (Ab7090, Abcam, 1:500) for 1 h at room temperature. An enhanced chemiluminescence kit was used to visualize the protein bands on the membrane, and the gray value of each band was analyzed using Image J software (NIH, Bethesda, MD, United States).

### Rat Model of IDD

Sprague–Dawley rats, aged 3 months, were used for the *in vivo* experiments. The rats were placed in a prone position after anesthesia with an intraperitoneal injection of 90 mg/kg ketamine and 10 mg/kg xylazine. Under fluoroscopic guidance, the IVDs were punctured with a 20-gauge needle from the dorsal side. The needle was inserted through the center of the disc to reach the opposite side, rotated 180°, and held for 10 s. After the surgery, the wound was covered with gauze, and a standard postoperative procedure was performed. One week after the initial puncture, a small incision was made to expose the previously punctured IVDs from the left side. A total of 2 μl solution containing a vector was slowly injected into the punctured discs using a 33-gauge needle (Hamilton, Bonaduz, Switzerland) attached to a microliter syringe (Hamilton). The injection procedure was repeated after 4 weeks.

### MRI Examination and Histological Evaluation

MRI was performed on all the rats using a 7.0 T animal-specific MRI system (Bruker Pharmascan, Ettlingen, Germany). The tissues were fixed in 4% paraformaldehyde for 48 h, decalcified in 10% EDTA, embedded in paraffin, and sectioned (5 μm) along the midsagittal plane. The sections were used for hematoxylin–eosin (HE) and safranin-O/fast green staining. Histological images were analyzed using a BX53 microscope (Olympus).

### Statistical Analysis

Quantitative data was shown as means ± standard deviation. One-way analysis of variance was used to evaluate the difference between the two groups. The correlation between circARL15, miR-431-5p, and DISC1 was analyzed by Pearson correlation analysis. A *p*-value of < 0.05 was used to denote statistical significance.

## Results

### Differentially Expressed circRNAs, lncRNAs, mRNAs, and miRNAs

**FIGURE 1:**
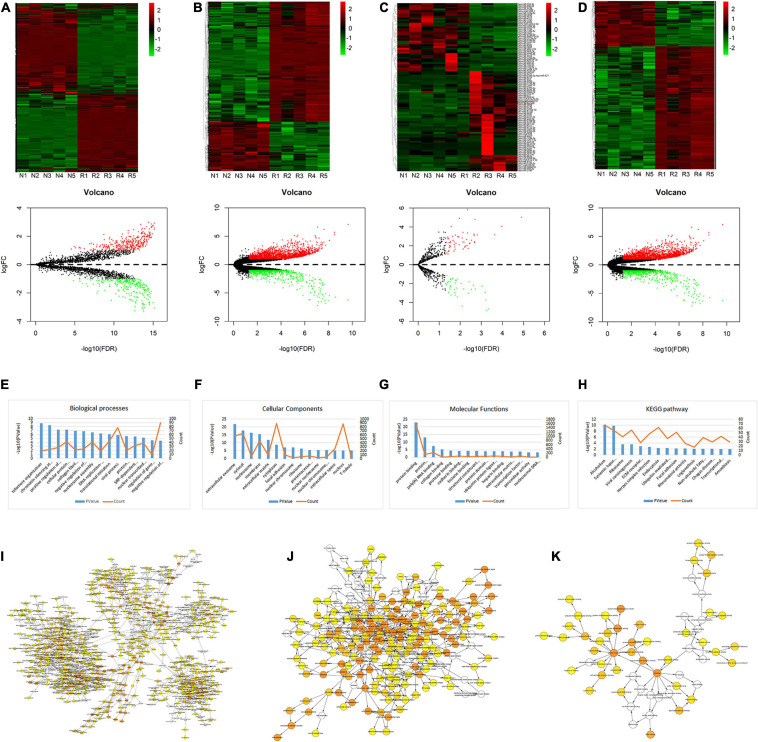
Heat map, volcano plots, GO annotation, and KEGG analysis of DEGs. **(A–D)** Heat map and volcano plots of circRNAs **(A)**, lncRNAs **(B)**, miRNAs **(C)**, and mRNAs **(D)**. The upper heat maps are based on expression values of significantly differentially expressed ncRNAs and mRNAs (absolute fold change ≥ 2.0 and *p* < 0.05) detected by microarray probes. “Red” and “Green” indicate expression above and below, respectively, relative expression. R = IDD group; N = control group. The bottom volcano plots reflect the number, significance, and reliability of differentially expressed ncRNAs and mRNAs. Red dots are up-regulated genes, green dots are down-regulated genes, and black dots are genes that were the same between the two groups. **(E–H)** GO annotation and KEGG analysis of differentially expressed mRNAs, with the top 15 enrichment scores covering domains of biological processes **(E)**, cellular components **(F)**, molecular functions **(G)**, and significant pathways **(H)**. Enrichment score values were calculated as –log 10 (*p*-values). **(I–K)** The BP **(I)**, CC **(J)**, and MF **(K)** were visualized in Cytoscape.

Gene expression dataset GSE67567 was downloaded from the GEO database. Microarray analysis was performed to investigate the DEGs in lumbar disk samples. Based on the cut-off criteria (fold change ≥ 2.0, *p* < 0.05, and FDR < 0.05), a total of 636 circRNAs, 2072 lncRNAs, 94 miRNAs, and 4416 mRNAs (Figures 1A–D) were found to be differentially expressed. Among the 636 differentially expressed circRNAs, 354 and 282 circRNAs were identified as up-regulated and down-regulated, respectively. Of the 2072 dys-regulated lncRNAs, 1,484 and 588 lncRNAs were identified as up-regulated and down-regulated, respectively. Additionally, 2,183 and 801 mRNAs were up-regulated and down-regulated, respectively, in the five degenerative lumbar disk samples, and the microarray expression profiles were found to contain 56 up-regulated miRNAs and 38 down-regulated miRNAs in the degenerated samples. The volcano plot was constructed to show the differences in these four transcripts between the two groups, through the *p*-value of the paired sample *t*-test and the fold-change values (Figures 1A–D). GO annotation and KEGG analysis of differentially expressed mRNAs were shown in Figures 1E–K.

### Construction of the ceRNA Network

**FIGURE 2:**
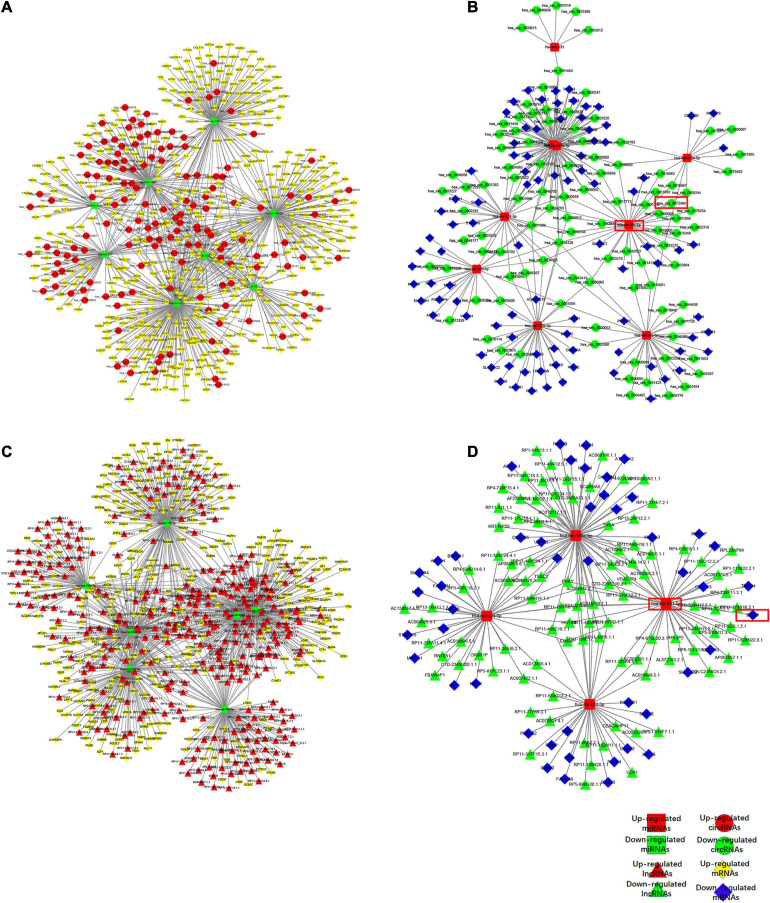
Competing endogenous RNA network in IDD. **(A,B)** The ceRNA network of down-regulated **(A)** or up-regulated **(B)** miRNA with circRNA and mRNA. **(C,D)** The ceRNA network of down-regulated **(C)** or up-regulated **(D)** miRNA with lncRNA and mRNA.

RNA transcripts can compete for the same MREs to communicate with each other by acting as ceRNAs, which indicated a complex relationship between the members of the ceRNA network. Based on our microarray data, we pioneered a ceRNA network in lumbar disk samples. We obtained 17 specific differentially expressed miRNAs using the methods described above to construct a network that highlighted the interaction between circRNAs and mRNAs, of which 8 were up-expressed miRNAs and nine were down-expressed miRNAs (Figures 2A,B). A total of 11 miRNAs were also included in the ceRNA network, demonstrating the association between lncRNAs and mRNAs (Figures 2C,D). Our findings revealed that the differentially expressed circRNAs and lncRNAs acted as ceRNAs to contact with mRNAs by competing for specific miRNAs. For example, hsa_circ_0072464 (circARL15), which was found to be the most differentially expressed circRNA within the ceRNA network, acted as an miR-431-5p sponge to modulate DISC1.

### Expression of circARL15, miR-431-5p, and DISC1 in IDD

**FIGURE 3:**
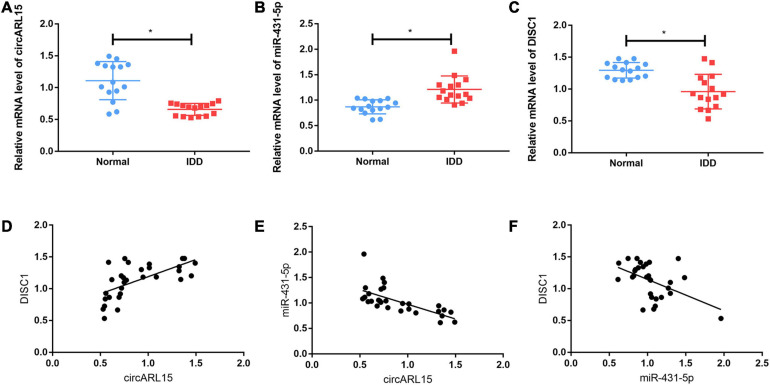
Expression of circARL15, miR-431-5p, and DISC1 in IDD. **(A–C)** The expressions of circARL15 **(A)**, miR-431-5p **(B)**, and DISC1 **(C)** in IDD and the normal samples. **(D–F)** circARL15 was positively correlated with DISC1 **(D)** and negatively correlated with miR-431-5p **(E)**, while miR-431-5p was negatively correlated with DISC1 **(F)** *p < 0.05.

circARL15, miR-431-5p, and DISC1 expressions of 30 NP tissues (15 from normal subjects and 15 from IDD subjects) were identified using quantitative real-time PCR. The results showed that circARL15 expression was decreased in the IDD group (Figure 3A), while the expression of miR-431-5p was increased (Figure 3B). Additionally, the expression of DISC1 was lower in the IDD subjects than in the normal controls (Figure 3C). All the expression levels were consistent with the results of GSE67567. However, the genes (IKBKAP, ISLR2, TSPYL4, GPR155, TBRG4, ADAM33, and SLC35E2) which could bind to miR-431-5p based on the ceRNA network were detected in IDD samples and had no differences between IDD and control samples (Supplementary Figures 1A–G). Next, we performed correlation analysis for these three genes. The results revealed that circARL15 was positively correlated with DISC1 and negatively correlated with miR-431-5p, while miR-431-5p was negatively correlated with DISC1 (Figures 3D–F). All these results demonstrated that circARL15, miR-431-5p, and DISC1 participate in the pathogenesis of IDD.

### circARL15 and DISC1 Bind Directly to miR-431-5p in IDD

**FIGURE 4:**
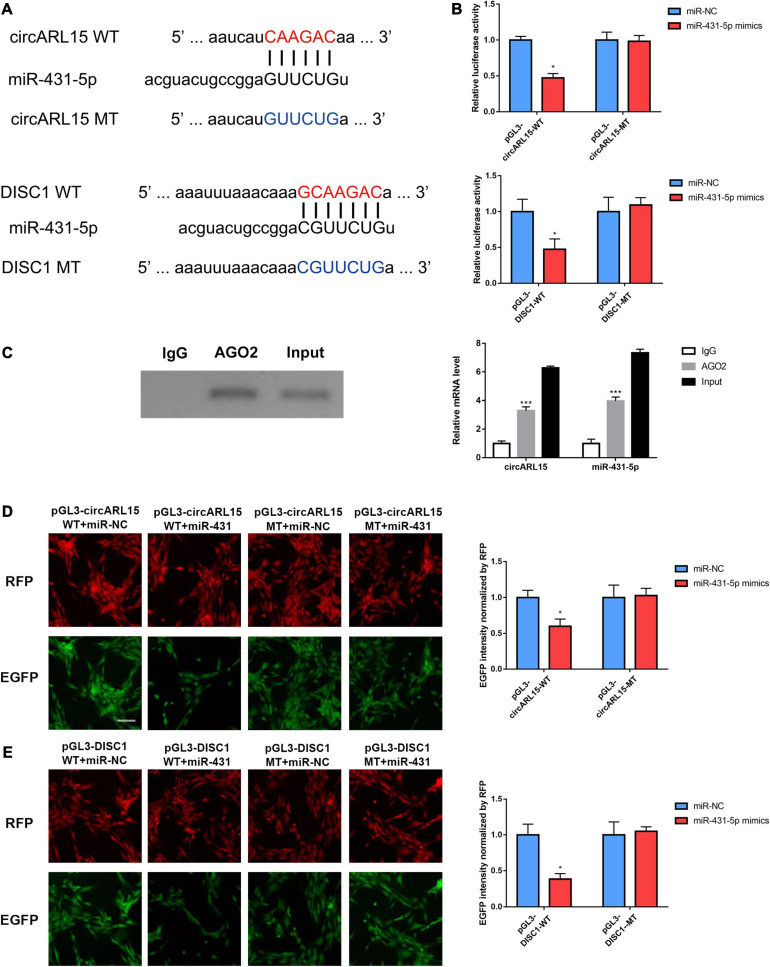
circARL15 and DISC1 bind directly to miR-431-5p in IDD. **(A)** The binding region of miRNAs in circARL15 and DISC1. **(B)** A luciferase reporter assay confirmed that miR-431-5p reduced the luciferase intensity in cells co-transfected with circARL15 WT and DISC1 WT. **(C)** RIP assay was used to detect the circARL15 and miR-431-5p mRNA levels (right). The AGO2 protein level was determined by Western blotting (left). **(D,E)** NP cells were co-transfected with an EGFP reporter plasmid or the mutant vector and the pDsRed-C1 plasmid, either alone or in combination with a miR-431-5p mimic. Forty-eight hours post-transfection, EGFP and RFP levels were measured *p < 0.05, ***p < 0.001.

The results of the bioinformatic analysis indicated that circARL15 served as an miR-431-5p sponge in the IDD subjects. Additionally, among the target genes of miR-431-5p, DISC1 was mostly decreased in the IDD tissues. To further validate the direct binding of miR-431-5p to circARL15 and DISC1, we constructed a plasmid with putative binding sites of circARL15 and DISC1 (Figure 4A). Next, luciferase reporter assays were used to further measure the direct binding of miR-431-5p to circARL15 and DISC1. After co-transfection with miR-431-5p mimic and luciferase reporters, the luciferase intensity of the NP cells was assessed. We showed that the luciferase intensity was reduced in miR-431-5p-overexpressed cells co-transfected with a circARL15 WT and DISC1 WT plasmid (Figure 4B). On the contrary, there was no significant difference in luciferase activity in cells co-transfected with miR-431-5p mimics and the mutated luciferase reporter (Figure 4B). Next, we performed RIP with an anti-AGO2 antibody and the results showed that circARL15 and miR-431-5p were significantly enriched, as they were precipitated by the anti-AGO2 antibody (Figure 4C).

In addition, to further confirm whether circARL15 and DISC1 could bind directly to miR-431-5p, an EGFP reporter assay was performed to determine the regulatory effects of miR-431-5p on circARL15 and DISC1. After inserting the WT and the MT sequence of circARL15 and DISC1 downstream of the EGFP-coding sequence, the circARL15 and DISC1 wild type and mutant vector were constructed. It was shown that cells co-transfected with miR-431-5p mimics and the WT reporter plasmid displayed suppressed EGFP expression. In contrast, co-transfection of miR-431-5p mimics with the mutated vectors showed little effect on EGFP expression (Figures 4D,E). All these findings provided evidence that miR-431-5p directly bound to circARL15 and DISC1, and indicated that circARL15 may be a ceRNA of DISC1 by harboring miR-431-5p in patients with IDD.

### Effects of circARL15 on Cell Apoptosis and Cell Proliferation

**FIGURE 5:**
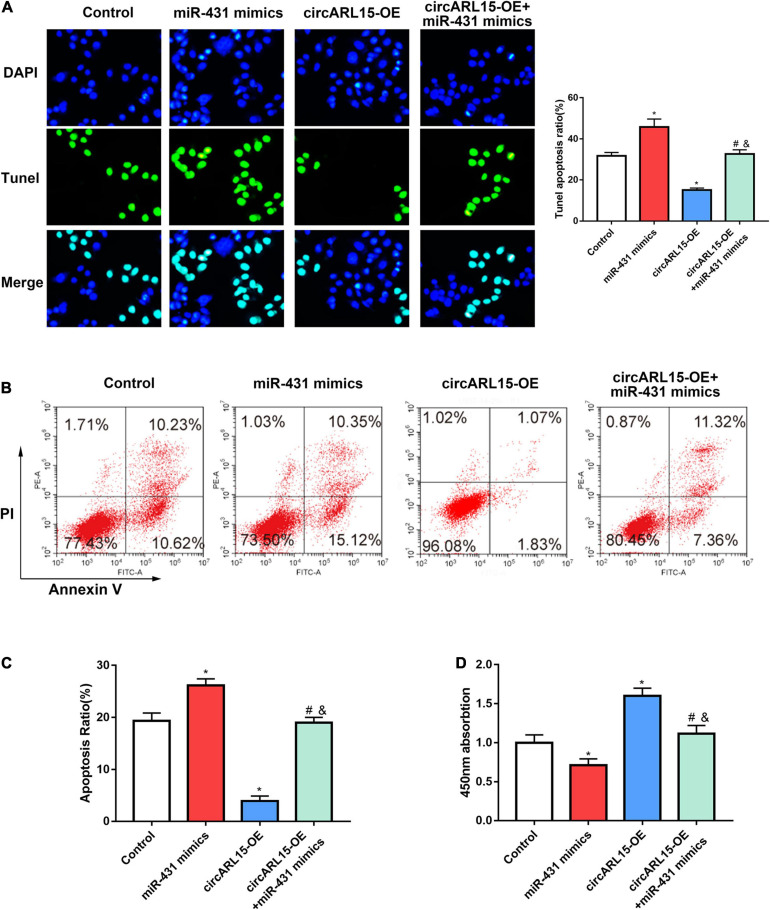
Effects of circARL15 on cell apoptosis and cell proliferation. NP cells were treated with negative control, circARL15 overexpression (OE) plasmid, miR-431-5p mimics, and circARL15-OE + miR-431-5p mimics. **(A)** Tunel assay of NP cells treated with different groups. **(B,C)** Cell flow cytometry was carried out to explore the cell viability of NP cells. **(D)** CCK-8 assay was performed in NP cells 48h after transfections. *p < 0.05 compared with control; ^#^*p* < 0.05 compared with miR-431-5p mimics. ^&^*p* < 0.05 compared with circARL15-OE.

In order to demonstrate whether circARL15 could modulate cell function through miR-431-5p, NP cells were subjected to the following treatments: negative control, a circARL15 overexpression (OE) plasmid, miR-431-5p mimics, and circARL15-OE + miR-431-5p mimics. The transfection efficiency was shown in Supplementary Figures 1H,I. A Tunel assay was performed to determine the effect of circARL15 on apoptosis in NP cells. It was shown that circARL15 overexpression suppressed cell apoptosis (*p* < 0.01), while miR-431-5p mimics revered the inhibition of circARL15 overexpression on cell apoptosis (Figure 5A). Similar results were observed by performing flow cytometry analysis on the cells (Figures 5B,C). A CCK-8 assay was used to examine the effect of circARL15 and miR-431-5p on cell proliferation, which demonstrated that circARL15 promoted NP cell proliferation, while cells co-transfected with circARL15-OE and miR-431-5p reversed this promotion (Figure 5D). These results revealed that circARL15 modulates NP cell function via its interaction with miR-431-5p.

### circARL15 Modulates the Expression of DISC1 via Its Interaction With miR-431-5p

**FIGURE 6:**
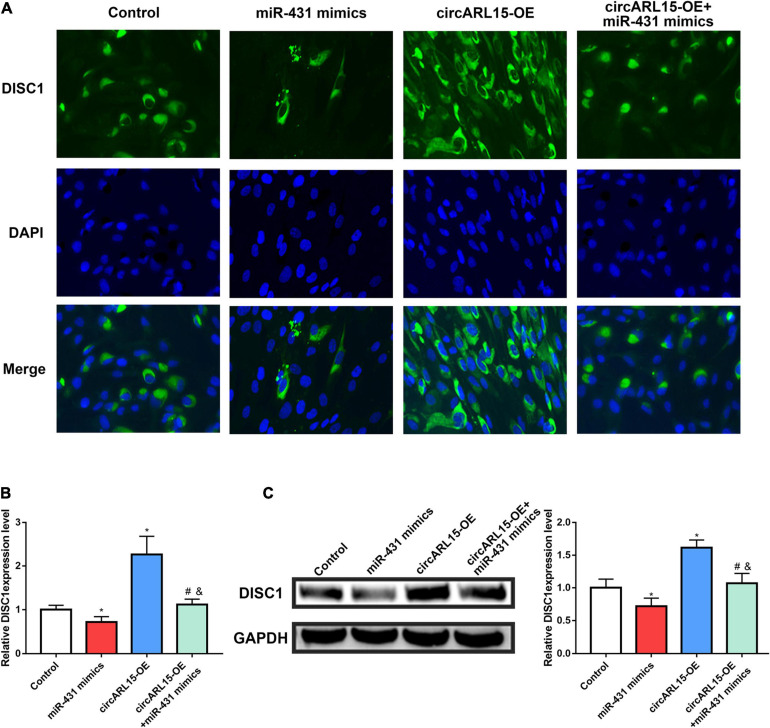
circARL15 modulates the expression of DISC1 through miR-431-5p. NP cells were treated with negative control, circARL15 overexpression (OE) plasmid, miR-431-5p mimics and circARL15-OE + miR-431-5p mimics. **(A,B)** Immunofluorescence staining of DISC1. **(C)** DISC1 protein expression levels were analyzed by western blot analysis. *p < 0.05 compared with control; ^#^*p* < 0.05 compared with miR-431-5p mimics. ^&^*p* < 0.05 compared with circARL15-OE.

We further accessed the regulation of circARL15 on DISC1 expression in NP cells. After treating NP cells with the following reagents, immunofluorescence analyses were performed: a negative control, circARL15 overexpression (OE) plasmid, miR-431-5p mimics, and circARL15-OE + miR-431-5p mimics. The immunofluorescence analyses results showed that circARL15-OE promoted the expression of DISC1, while this effect was reduced by co-transfection with circARL15-OE and miR-431-5p mimics (Figures 6A,B). Additionally, Western blot analysis showed that the protein level of DISC1 was increased by circARL15-OE and that this promotion could be mitigated by co-transfection of circARL15-OE and miR-431-5p mimics (Figure 6C). These results showed that circARL15 modulates the expression of DISC1 via its interaction with miR-431-5p.

### Intradiscal Injection of the circARL15 Alleviated IDD *in vivo*

**FIGURE 7:**
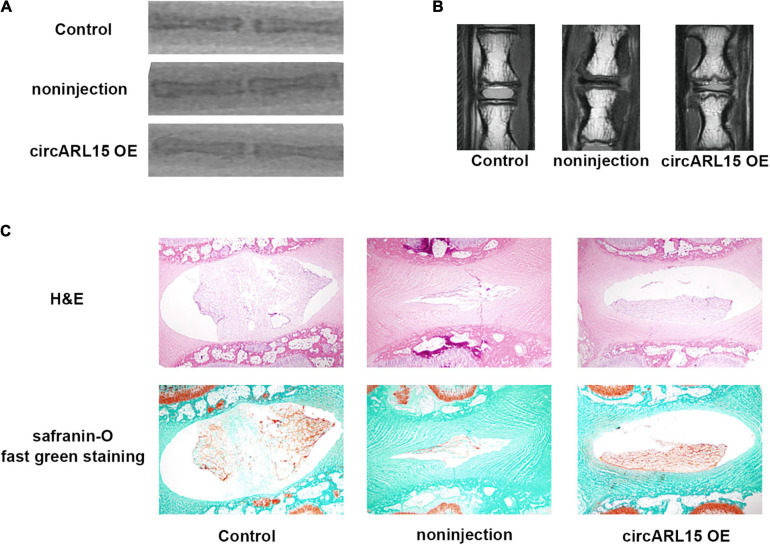
Intradiscal injection of the circARL15 alleviates IDD *in vivo*. **(A)** Radiographs of the indicated groups were obtained 9 weeks after needle puncture. **(B)** MRIs of the indicated groups were obtained 9 weeks after needle puncture. **(C)** H&E and safranin-O/fast green staining of the IVDs in the indicated groups.

We established a rat model of IDD through needle puncture. At 1 and 5 weeks after puncture, the circARL15 over-expression plasmid was injected into the punctured IVDs. X-rays were conducted to demonstrate progressive disc space narrowing over time in all the IVD puncture groups (Figure 7A). After injection, the MRI showed that degeneration of the IVDs was rescued in the circARL15 over-expression plasmid group than in the non-injection group (Figure 7B). The injection of the circARL15 over-expression plasmid alleviated the loss of NP tissue and the destruction of disc structures (Figure 7C). These results further validated the critical function of circARL15 *in vivo*.

## Discussion

Intervertebral disk degeneration is the major cause of lower back pain in adults (Ruan et al., 2007; Yu et al., 2020). Using the tissues of IDD patients, the present study aimed to identify the DEGs involved in IDD by analyzing a set of previously published microarray data (Lan et al., 2016). In this study, we identified 354 up-regulated and 282 down-regulated circRNAs, 1,484 up-regulated and 588 down-regulated lncRNAs, and 2,183 up-regulated and 801 down-regulated mRNAs, which were revealed to be significantly differentially expressed in IDD patient tissue samples. Some of the DEGs were shown to participate in several vital biological IDD processes. One previous research showed that miRNA 155 promoted cell apoptosis in progressive IDD by inducing FADD (Fas-associated death domain) and caspase-3 (Wang et al., 2011). Furthermore, lncRNA TUG1 was demonstrated to inhibit apoptosis in TNF-α–treated neural precursor cells and promote cell proliferation, possibly by targeting the Wnt/β-catenin signaling pathway (Chen et al., 2017). Though much has been done to clarify the role of DEGs in the progression of this disease (Ma et al., 2016), much still remains to be uncovered to better understand the pathogenesis of IDD.

Gene ontology analysis showed that IDD may be associated with certain biological processes and molecular functions. It is generally accepted that the destruction of ECM plays a critical role in the development of IDD. Degradation of ECM helps endothelial cells to migrate to further induce the generation of neovascularization, which later leads to the occurrence of IDD (Chen et al., 2017). In IDD, circRNAs were found to participate in the biological processes of protein sumoylation, and protein transport may provide us a perspective to understand the disease pathogenesis. The KEGG (Kyoto Encyclopedia of Genes and Genomes) database was used for analysis purposes to determine the pathways associated with the DEGs involved in the pathogenesis mechanisms of IDD. Hippo signaling pathway, tight junction, adherens junction, endocytosis were the four enriched pathways of the differentially expressed lncRNAs were the Hippo signaling pathway, tight junction pathway, adherens junction pathway, and endocytosis pathway. Differentially expressed circRNAs were shown to be enriched in 15 pathways such as the ubiquitin-mediated proteolysis pathway, AMPK (adenosine monophosphate–activated protein kinase) signaling pathway, and Wnt signaling pathway (Cully, 2013), which are closely correlated with the pathogenesis of IDD. As its upstream regulator of autophagy, AMPK was shown to exert a protective effect against apoptosis of NP cells (Chen et al., 2016). Several researches have suggested that Wnt/β-catenin signaling plays an important role in IDD, and our findings were in accordance with the prior researches (Chen et al., 2016). Dysregulated mRNAs were found to be enriched in ribosome, viral carcinogenesis, ECM–receptor interaction, herpes simplex infection, endocytosis, ubiquitin-mediated proteolysis, focal adhesion, and rheumatoid arthritis, which may be possible pathogenic pathways in IDD.

In addition, our study provided fresh insight into the modulated expression of 17 circRNAs and 11 lncRNAs, which function as ceRNAs in this degenerative disease. Among them, circARL15 is the most differentially expressed circRNA in the ceRNA network, according to the findings of our bioinformatics analysis. We also found that circARL15 could act as an miR-431-5p sponge to modulate DISC1 in IDD. Increasing evidence indicates that circRNAs participates in the pathogenesis of IDD, acting as a ceRNA. CircularRNA_104670 has been shown to play a critical role in IDD by functioning as a ceRNA of miR-17-3p (Song et al., 2018). It has also been shown that circ-FAM169A promotes IDD progression by targeting the miR-583/BTRC signaling pathway to regulate ECM catabolism and anabolism (Guo et al., 2020). This study confirmed that circARL15 modulates the apoptosis and proliferation of NP cells. Additionally, circARL15 was found to regulate the expression of DISC1 by sponging miR-431-5p. Also, miR-431-5p was demonstrated to bind directly to circARL15 and DISC1 in NP cells. In terms of the role of miR-431-5p in diseases, down-regulation of this miRNA has been reported to promote cell proliferation by targeting LRSAM1 in Hirschsprung’s disease (Hu et al., 2019). miR-431 has also been shown to accelerate cell proliferation and inhibit cell apoptosis, and to be associated with the lung developmental disorders (Li S. et al., 2019). In our study, miR-431-5p was significantly increased in IDD and overexpression of miR-431-5p suppressed cell proliferation and increased cell apoptosis, which were the effects of transfecting NP cells with a circARL15 overexpression plasmid. DISC1, which encodes a protein with multiple coiled-coil motifs, is acknowledged to play a regulatory role in neurite outgrowth and cortical development. DISC1 has been reported to be regulated by microRNA-TF to modulate neuronal migration via an *in silico* approach (John et al., 2019). However, whether DISC1 could be regulated by some other ncRNAs is largely unknown. Here, DISC1 was decreased in IDD and was positively related with circARL15. We also provided evidence that the expression of DISC1 was regulated by circARL15 via its interaction with miR-431-5p.

In conclusion, our study analyzed and identified the IDD-specific ncRNA profiles, of which circARL15 was found to be down-regulated. circARL15 regulated DISC1 by directly sponging miR-431-5p to regulate NP cell proliferation and apoptosis. Our data may provide a prospective therapeutic target in the treatment of IDD.

## Data Availability Statement

Publicly available datasets were analyzed in this study. This data can be found here: GSE67567.

## Ethics Statement

The studies involving human participants were reviewed and approved by the Ethics Committee of Fuyang Hospital of Anhui Medical University. The patients/participants provided their written informed consent to participate in this study.

## Author Contributions

HW and HF conceived and designed the study. YZ and WQ performed the study. LC and ZG analyzed the data. HF and KS wrote the manuscript. All authors discussed the results and commented on the manuscript.

## Conflict of Interest

The authors declare that the research was conducted in the absence of any commercial or financial relationships that could be construed as a potential conflict of interest.

## Footnotes

1 http://www.ncbi.nlm.nih.gov/geo/

## Supplementary Material

Supplementary Figure 1 **(A–G)** The mRNA level of IKBKAP, ISLR2, TSPYL4, GPR155, TBRG4, ADAM33 and SLC35E2 in IDD and control samples. **(H,I)** The transfection efficiency of miR-431-5p mimics and circARL15 over-expression plasmid. ****P* < 0.001.
