# Supplementary figures and images for: circARL15 Plays a Critical Role in Intervertebral Disc Degeneration by Modulating miR-431-5p/DISC1
Source: Front Genet. 2021 Jun 21;12:669598. doi: 10.3389/fgene.2021.669598 (PMC8255806; doi:10.3389/fgene.2021.669598)

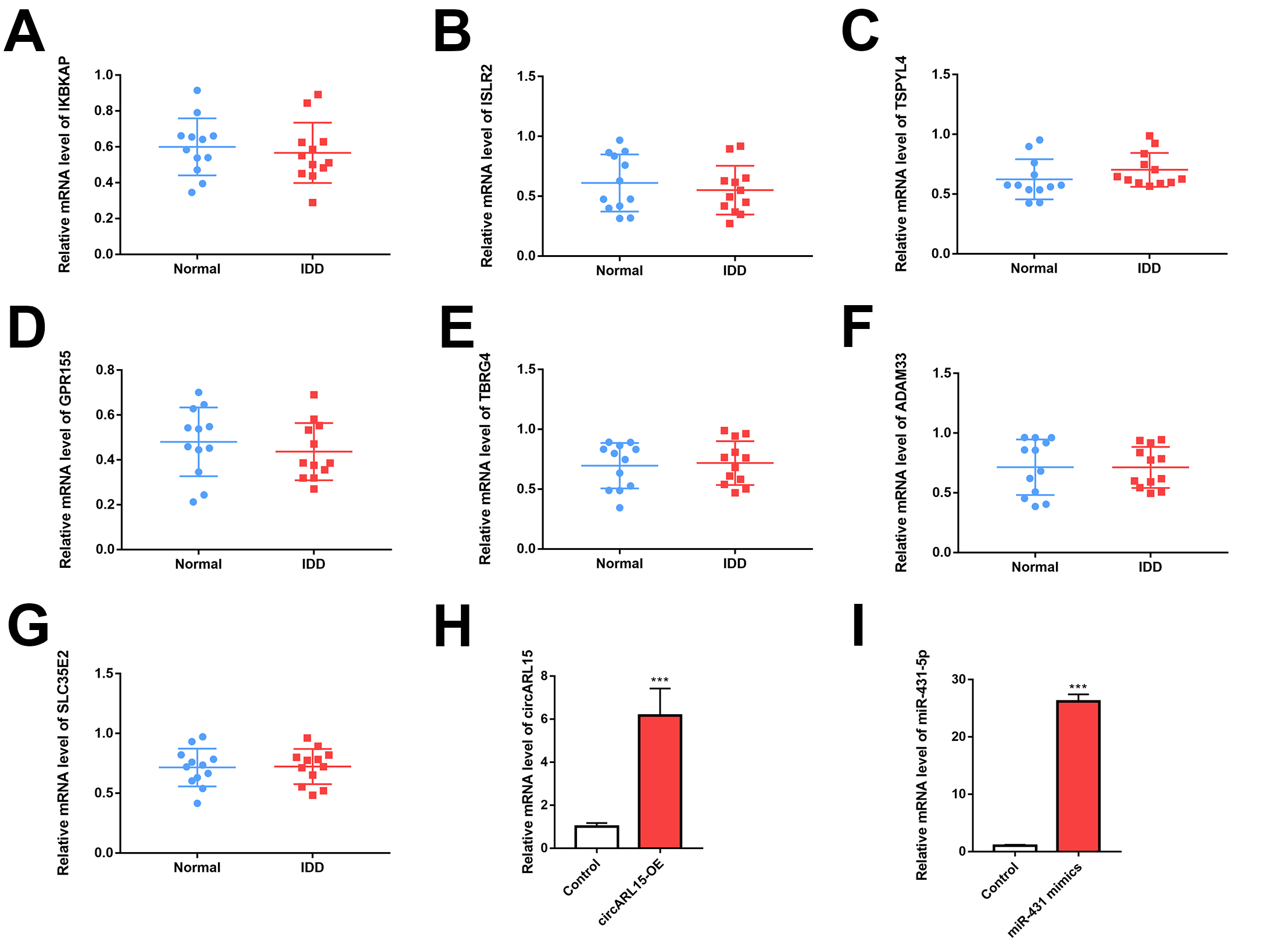

Supplement: Supplementary Figure 1 — (A–G) The mRNA level of IKBKAP, ISLR2, TSPYL4, GPR155, TBRG4, ADAM33 and SLC35E2 in IDD and control samples. (H,I) The transfection efficiency of miR-431-5p mimics and circARL15 over-expression plasmid. ***P < 0.001. [file Image_1.TIF]
